# A LuxR‐type regulator, AcrR, regulates flagellar assembly and contributes to virulence, motility, biofilm formation, and growth ability of *Acidovorax citrulli*

**DOI:** 10.1111/mpp.12910

**Published:** 2020-01-14

**Authors:** Wei Guan, Tielin Wang, Qi Huang, Eryuan Tian, Bo Liu, Yuwen Yang, Tingchang Zhao

**Affiliations:** ^1^ State Key Laboratory for Biology of Plant Diseases and Insect Pests Institute of Plant Protection Chinese Academy of Agricultural Sciences Beijing China; ^2^ State Key Laboratory Breeding Base of Dao‐di Herbs National Resource Center for Chinese Materia Medica China Academy of Chinese Medical Sciences Beijing China; ^3^ Floral and Nursery Plants Research Unit U.S. Department of Agriculture Agricultural Research Service Beltsville MD USA

**Keywords:** *Acidovorax citrulli*, AcrR, flagellar assembly, LuxR‐type regulator

## Abstract

LuxR‐type regulators regulate many bacterial processes and play important roles in bacterial motility and virulence. *Acidovorax citrulli* is a seedborne bacterial pathogen responsible for bacterial fruit blotch, which causes great losses in melon and watermelon worldwide. We identified a LuxR‐type, nonquorum sensing‐related regulator, AcrR, in the group II strain Aac‐5 of *A. citrulli*. We found that the *acrR* mutant lost twitching and swimming motilities, and flagellar formation. It also showed reduced virulence, but increased biofilm formation and growth ability. Transcriptomic analysis revealed that 394 genes were differentially expressed in the *acrR* mutant of *A. citrulli*, including 33 genes involved in flagellar assembly. Our results suggest that AcrR may act as a global regulator affecting multiple important biological functions of *A. citrulli.*

## Graphical abstract

AcrR, a LuxR‐type regulator, acts as a global regulator affecting multiple important biological functions, especially swimming motility and flagellum assembly of *Acidovorax citrulli.*

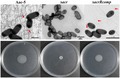

## 1 INTRODUCTION

Transcriptional regulators of the LuxR family have been found in a wide range of bacteria, where they regulate various bacterial functions, including plasmid conjugation, antibiotic production, cell motility, and sporulation (Pappas and Winans, 2003; Kuwana *et al.*, 2004; Qian *et al.*, 2013; Singer *et al.*, 2013). These regulators are approximately 250 amino acids in size with a helix‐turn‐helix (HTH) DNA‐binding domain at the C‐terminus, and a variable domain at the N‐terminus that interacts with signalling substances, for example quorum‐sensing, two‐component signalling transduction, and other signals (Patankar and González, 2009; Santos *et al.*, 2012). LuxR is best known for its role in quorum sensing (QS), which bacteria use to communicate with each other, and often pairs with LuxI, an autoinducer synthase protein. In addition to QS, LuxR‐type proteins are also involved in other cellular signalling pathways. Several *luxR* genes not associated with a *luxI* gene are responsible for modulating the expression of virulence factors, biofilm formation, and host immune responses (Fuqua *et al.*, 2001; Santos *et al.*, 2012). In *Salmonella enterica*, a LuxR‐type transcriptional regulator, RflM, harbours a conserved HTH domain at the C‐terminal, whereas it does not contain any domain at the N‐terminal. RflM negatively regulates the expression of *flhDC*, which encodes FlhD_4_C_2_, the flagellar master regulatory complex, which, in turn, affects the flagellar assembly (Singer *et al.*, 2013).

Bacterial fruit blotch is a seedborne disease that causes significant yield losses in melon and watermelon worldwide. Its causal agent is *Acidovorax citrulli*, a gram‐negative bacterium with a single polar flagellum (Bahar and Burdman, 2010). Based on DNA‐fingerprinting profiles, whole‐cell fatty‐acid composition, carbon‐source utilization, pathogenicity assays, pulsed‐field gel electrophoresis, and multilocus sequence typing, strains of *A. citrulli* are divided into two groups: group I includes strains isolated mainly from nonwatermelon hosts and are pathogenic to most cucurbit hosts, and group II is composed of strains isolated mainly from watermelon and more aggressive to watermelon than the group I strains (Walcott *et al.*, 2004; Burdman *et al.*, 2005; Feng *et al.*, 2009; Yan *et al.*, 2013). The role of QS in *A. citrulli* has been studied in both the group I strain XJL12 and the group II strain Aac‐5 (Chen *et al.*, 2009; Wang *et al.*, 2016). Mutants of both the *luxI*/*luxR* and *aacI*/*aacR* showed reduced virulence and motility, indicating the important role QS plays in *A. citrulli* (Chen *et al.*, 2009; Wang *et al.*, 2016).

In addition to *luxR* and *aacR*, many other LuxR‐type regulators in the genome of *A. citrulli* have been identified in our preliminary studies (data not shown). In this study, we explored the regulatory role of one of the LuxR‐type regulators, AcrR, in the group II strain Aac‐5 of *A. citrulli* by mutational analysis and RNA sequencing. Our results showed that deletion of *acrR* affected flagellar biosynthesis, cell motility, biofilm formation, and virulence of Aac‐5 of *A. citrulli*. In addition, our RNA sequencing (RNA‐Seq) results revealed that the expression of many genes involved in various functions of strain Aac‐5 of *A. citrulli*, including flagellar assembly, were changed in *acrR* deletion mutant strain, further suggesting that *acrR* functions as a global transcriptional regulator, especially for flagella‐related functions in *A. citrulli*.

## 2 RESULTS

### 2.1 Identification of AcrR, a putative LuxR‐type regulator of *A. citrulli,* and generation of *acrR* mutant and complemented strains

An open reading frame (ORF) containing 209 amino acids was identified in the genome sequence of the *A. citrulli* group II strain AAC00‐1 (GenBank accession number CP000512.1), located from nucleotide 4874913 to 4875542, with the locus tag of Aave_4382. It has a typical domain structure of the LuxR‐family response regulators: a receiver domain at the N‐terminus and an HTH DNA‐binding domain at the C‐terminus. In addition, a BLASTp search showed that this ORF has 100% amino acid sequence identity with the protein ADX48163.1 in *Acidovorax avenae* subsp. *avenae* ATCC 19860, annotated as a two‐component transcriptional regulator in the LuxR family. The *acrR* has no similarities with the quorum‐sensing signal receptor *aacR* (located from nucleotide 4232080 to 4232808, with the locus tag of Aave_3810 in genome of AAC00‐1, Wang *et al.*, 2016) at the nucleotide or protein level. This suggests that the ORF in *A. citrulli* strain AAC00‐1 is also a LuxR‐type transcriptional regulator and was designated AcrR because it is an *A. citrulli* regulator that is different from *aacR* in the group II strain Aac‐5 of *A. citrulli*.

The successful construction of the *acrR* mutant strain ∆*acrR* was confirmed by PCR amplification of strain ∆*acrR* with the LacrR‐F and RacrR‐R primers (Table 1) and subsequent sequencing of the PCR product. The product is 1,868 bp in size, containing 472‐bp upstream and 541‐bp downstream fragments of the *acrR* gene, separated by an 855‐bp gentamicin cassette (data not shown). This was different from the PCR product of 1,643 bp in size that was amplified from the wild‐type strain Aac‐5, which contained the 630‐bp *acrR* gene as well as the same 472‐bp upstream and 541‐bp downstream sequences.

**Table 1:** Primers used for construction of mutant and complemented strains

Primers	Sequence (5′–3′, restriction enzyme sites are underlined)	Restriction enzyme	Product of PCR (bp)
LacrR‐F	GAATTCCGAGCCAGCGCATCAT	*Eco*RI	472
LacrR‐R	GGATCCGGCAATTCTCCTGGGTC	*Bam*HI	
RacrR‐F	GTCGACCGCCCCGCCGTCCTAT	*Sal*I	541
RacrR‐R	AAGCTTCATGTCGGTCGGCTTCG	*Hin*dIII	
acrR‐F	CCCAAGCTTATGATCCACGTCGTGC	*Hin*dIII	630
acrR‐R	CGGGATCCTCACACCAGCTGGTTG	*Bam*HI	
GmF	GGATCCCGACGCACACCGTGGAAA	*Bam*HI	855
GmR	GTCGACGCGGCG TTGTGACAATTT	*Sal*I	

The complementation strain ∆*acrR*comp showed resistance to kanamycin, suggesting the successful transfer of the plasmid pBBR‐acrR into ∆*acrR* (Table 2). The presence of pBBR‐acrR in ∆*acrR*comp was further confirmed by PCR amplification of the strain ∆*acrR*comp with the primers acrR‐F and acrR‐R (Table 1) and sequencing of the PCR product, which showed the PCR amplified the 630‐bp *acrR* gene.

**Table 2:** Bacterial strains and plasmids used in this study

Strain or plasmid	Description	Reference or source
*Strains*		
*Escherichia coli*		
DH5α	*supE44* Δ*lacU169*(Φ80*lacZ* Δ*M15*) *hsdR17 recA1 endA1 gyrA96 thi‐1 relA1*	Hanahan, (1983)
*Acidovorax citrulli*		
Aac‐5	Wild‐type watermelon strain; Ap^r^	Yan *et al. *(2013)
Δ*acrR*	*acrR* mutant, Ap^r^; Gm^r^	This study
Δ*acrR*comp	Δ*acrR* complementation strain, Δ*acrR* containing pBBR‐acrR; Ap^r^, Gm^r^, Km^r^	This study
*Plasmids*		
pK18mobsacB	Cloning and suicide vector with *sacB* for mutagenesis; Km^r^	Schäfer *et al. *(1994)
pK18‐acrR‐Up&Down	pK18mobsacB carrying 472‐ and 541‐bp upstream and downstream sequences of *acrR*; Km^r^	
pK18‐*acrR*Gm	pK18mobsacB carrying 630‐bp coding region of *acrR* replaced by 855‐bp Gm cassette, as well as 472‐ and 541‐bp upstream and downstream sequences of the gene; Km^r^	This study
pBBR1MCS‐2	Broad‐host range expression vector; Km^r^	Kovach *et al. *(1994)
pBBR‐acrR	pBBR1MCS‐2 carrying 630‐bp coding region of *acrR*; Km^r^	This study

Note

Km^r^, Ap^r^, and Gm^r^ indicate resistance to kanamycin, ampicillin, and gentamicin, respectively.

### 2.2 The *acrR* mutant of *A. citrulli* was reduced in virulence

**Figure 1:**
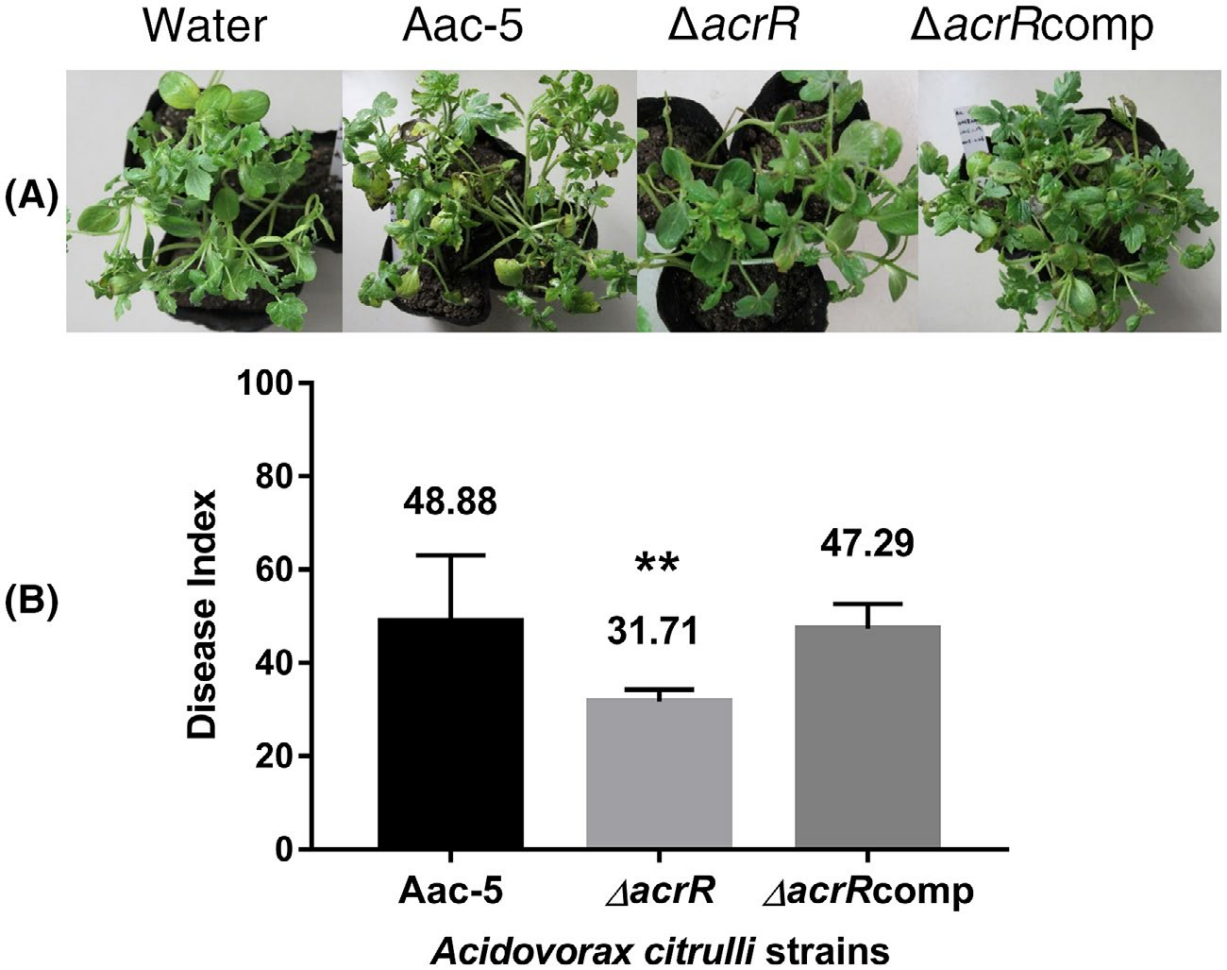
Effect of *acrR* on virulence of *Acidovorax citrulli* on watermelon leaves. (a) Appearance of watermelon seedlings 10 days after inoculation with sterile water, the wild‐type strain Aac‐5, the *acrR* mutant strain ∆*acrR*, and the mutant complementation strain ∆*acrR*comp of *A. citrulli*. (b) Virulence of *A. citrulli* strains 10 days after inoculation, calculated based on a disease index of 0 to 100 (Wang *et al*., 2016). The bars represent standard errors of the means from three experiments, each containing six inoculated watermelon seedlings in three pots per tested strain. **p < .01 by Student's *t* test

To investigate whether AcrR contributes to the virulence of *A. citrulli*, we compared the virulence of the wild‐type strain Aac‐5 in watermelon seedlings with the mutant strain ∆*acrR* and the complementation strain ∆*acrR*comp. Ten days after inoculation, the disease index (DI) in the watermelon seedlings that was caused by the mutant strain ∆*acrR* was 31.71, which was significantly lower than the DI of 48.88 caused by the wild‐type strain Aac‐5 (*p* < .01). The DI caused by the complementation strain ∆*acrR*comp was 47.29, similar to the wild‐type strain Aac‐5 (Figure 1).

**Figure 2:**
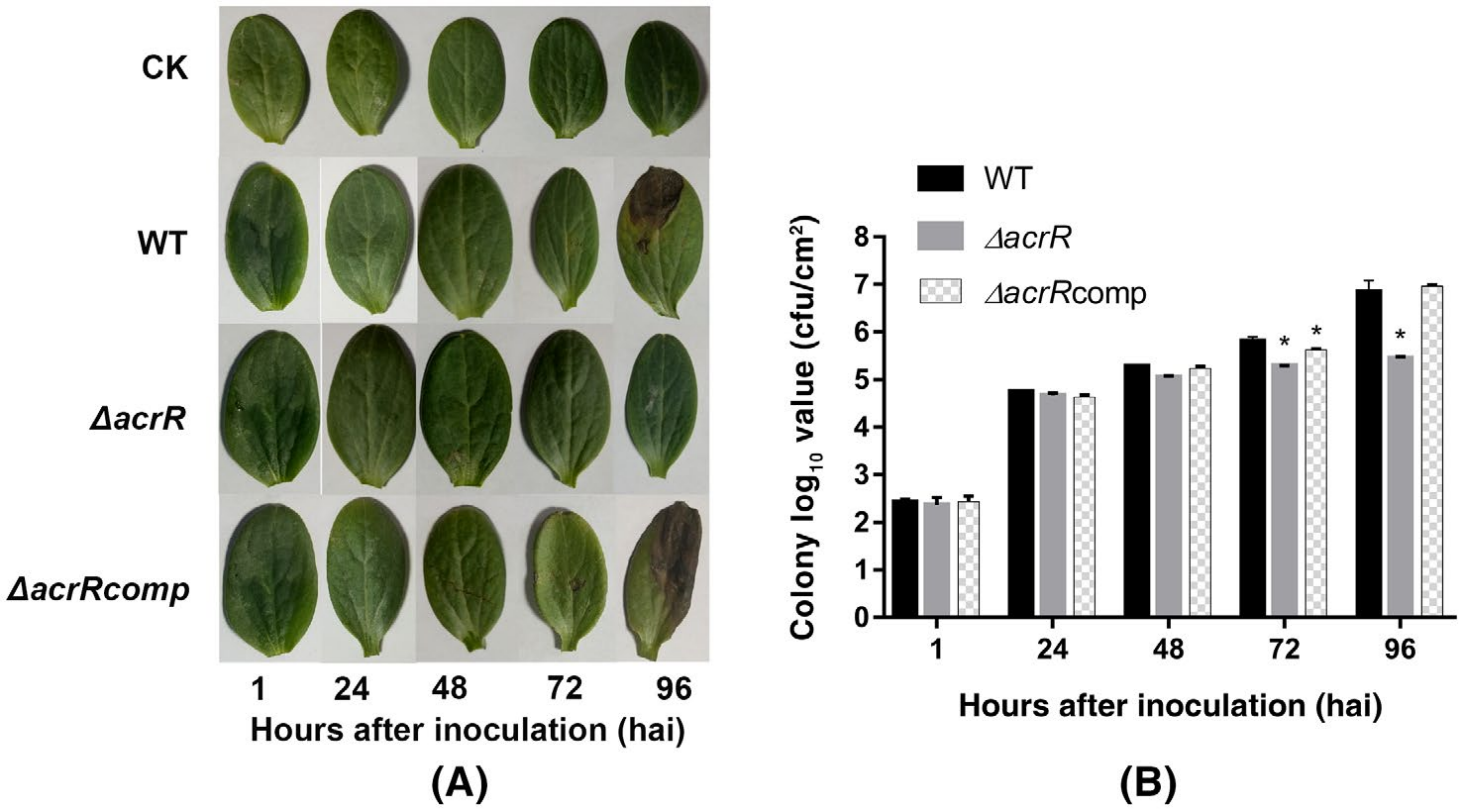
Effect of *acrR* on growth ability of *Acidovorax citrulli* in watermelon cotyledons. (a) Appearance of watermelon cotyledons inoculated with sterile water (CK), the wild‐type strain Aac‐5 (WT), the *acrR* mutant strain ∆*acrR* (∆*acrR*), and the mutant complementation strain ∆*acrR*comp (∆*acrR*comp) of *A. citrulli* at 1, 24, 48, 72 and 96 hr after inoculation (hai). (b) Count of colonies isolated from leaf disks of watermelon cotyledons inoculated with the WT, ∆*acrR*, and ∆*acrR*comp strains 1, 24, 48, 72 and 96 hai. The bars represent standard errors of the means from three experiments, each containing six cotyledon discs per stain per time point. The asterisks indicate a significant difference compared to the WT strain, calculated by Student's *t* test (*p* < .05)

To determine whether the mutant is defective in its ability to grow in planta, we injected watermelon cotyledons with bacterial cell suspensions of the wild‐type strain Aac‐5, the mutant strain ∆*acrR*, and the complementation strain ∆*acrR*comp, as well as sterile water as a negative control. No symptoms were observed in inoculated cotyledons 1 and 24 hr after inoculation (hai) in all treatments, whereas water‐soaking necrosis appeared 48 hai, and lesions started to develop 72 and 96 hai (Figure 2a) in cotyledons inoculated with the wild‐type and complementation strains, but not in those inoculated with water and the mutant strain. The results from our quantitative bacterial in planta assay revealed that the populations of the Aac‐5, the ∆*acrR*, and the ∆*acrR*comp strains in cotyledons were not significantly different until 72 hai, when the populations of the ∆*acrR* and the ∆*acrR*comp strain were significantly lower than that of the Aac‐5 strain (Figure 2b). At 96 hai, the population of the ∆*acrR* strain remained similar to its population level at 72 hai but was significantly lower than that of the Aac‐5 and the ∆*acrR*comp strains (Figure 2b).

### 2.3 Biofilm formation and growth rate of the *acrR* mutant of *A. citrulli* were increased 

**Figure 3:**
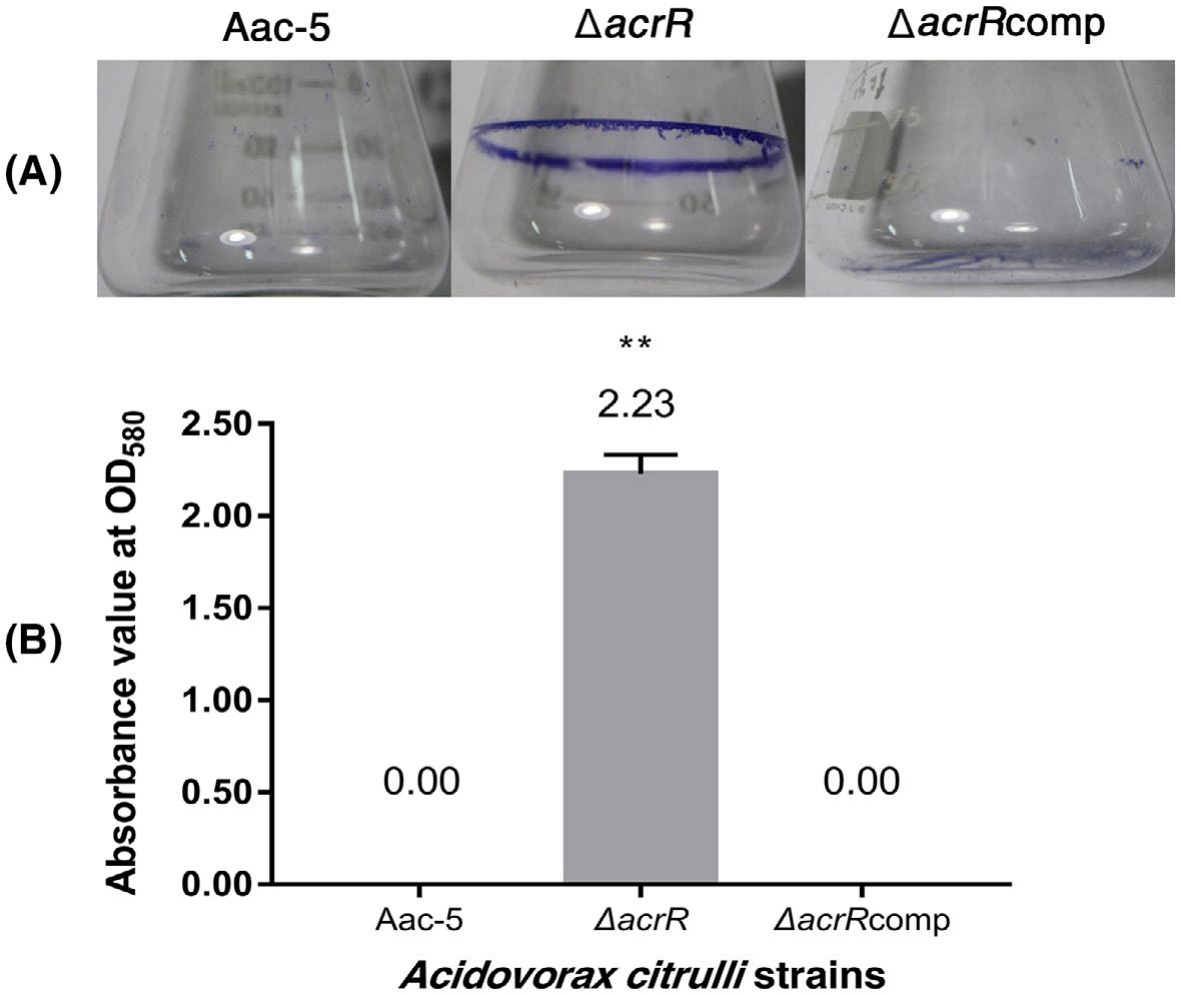
Effect of *acrR* on biofilm formation in *Acidovorax citrulli*, as visualized by the violet ring formed on the inner surface of the glass flasks (a) and measured under OD_580_ using stained biofilm solubilized with ethanol (b). The error bar represents standard errors of the means from three experiments, each containing three replicates per treatment. **p < .01 by Student's *t* test. Aac‐5, wild‐type strain of *A. citrulli*; ∆*acrR*, *acrR* deletion mutant of Aac‐5; ∆*acrR*comp, *acrR* complemented strain of ∆*acrR*

The wild‐type strain Aac‐5 did not form any biofilm when measured both qualitatively and quantitatively in our study (Figure 3). When the *acrR* gene was mutated, however, the mutant strain ∆*acrR* formed a visible ring of biofilm on the inner wall of a flask, while no such ring was observed for the complementation strain ∆*acrR*comp (Figure 3a). This observation was confirmed by our quantitative biofilm assay, since the mean absorption value of the biofilm by ∆*acrR* was 2.23, while the absorption was below the detection levels for the wild‐type strain Aac‐5 and the complementation strain ∆*acrR*comp (Figure 3b). Our results showed that the mutation of the *acrR* gene enhanced the biofilm formation of *A. citrulli*.

**Figure 4:**
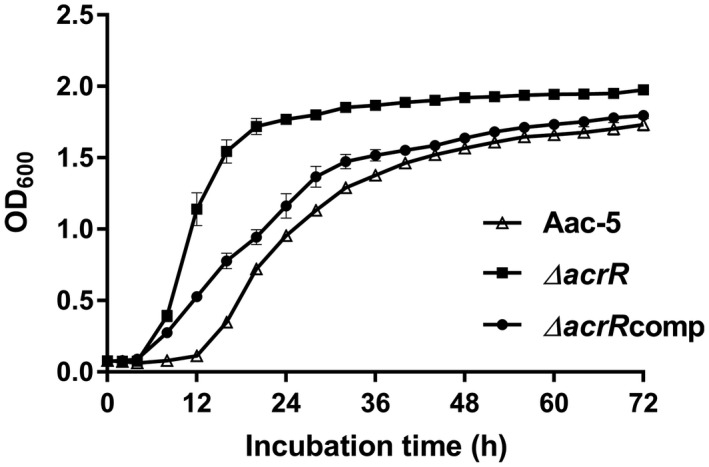
Effect of *acrR* on growth ability of *Acidovorax citrulli* in liquid King's B medium. Optical density (OD_600_) of the cultured cell suspensions of the tested *A. citrulli* strains measured at time points of 0–72 hr. The error bar represents standard errors of the means from three experiments, each containing five replicates per strain. Aac‐5, wild‐type strain of *A. citrulli*; ∆*acrR*, *acrR* deletion mutant of Aac‐5; ∆*acrR*comp, *acrR* complemented strain of ∆*acrR*

The growth ability of Aac‐5, ∆*acrR*, and its complementation strain ∆*acrR*comp was determined by measuring the optical density of cell suspensions incubated in King's B broth at 28 °C. The mutant strain ∆*acrR* was increased in growth ability, with the OD_600_ value reaching 1.14 at 12 hr of incubation, and in the meantime the OD_600_ value of the complementation strain ∆*acrR*comp reached 0.53, whereas the wild‐type strain Aac‐5 had an OD_600_ value of 0.11 at the same time point. When incubated for 36 hr, the OD_600_ value of ∆*acrR* was 1.87, while those of the Aac‐5 and ∆*acrR*comp were 1.38 and 1.52, respectively (Figure 4).

### 2.4 The *acrR* mutant of *A. citrulli* lost the ability to twitch and swim, as a result of the loss of flagella formation

**Figure 5:**
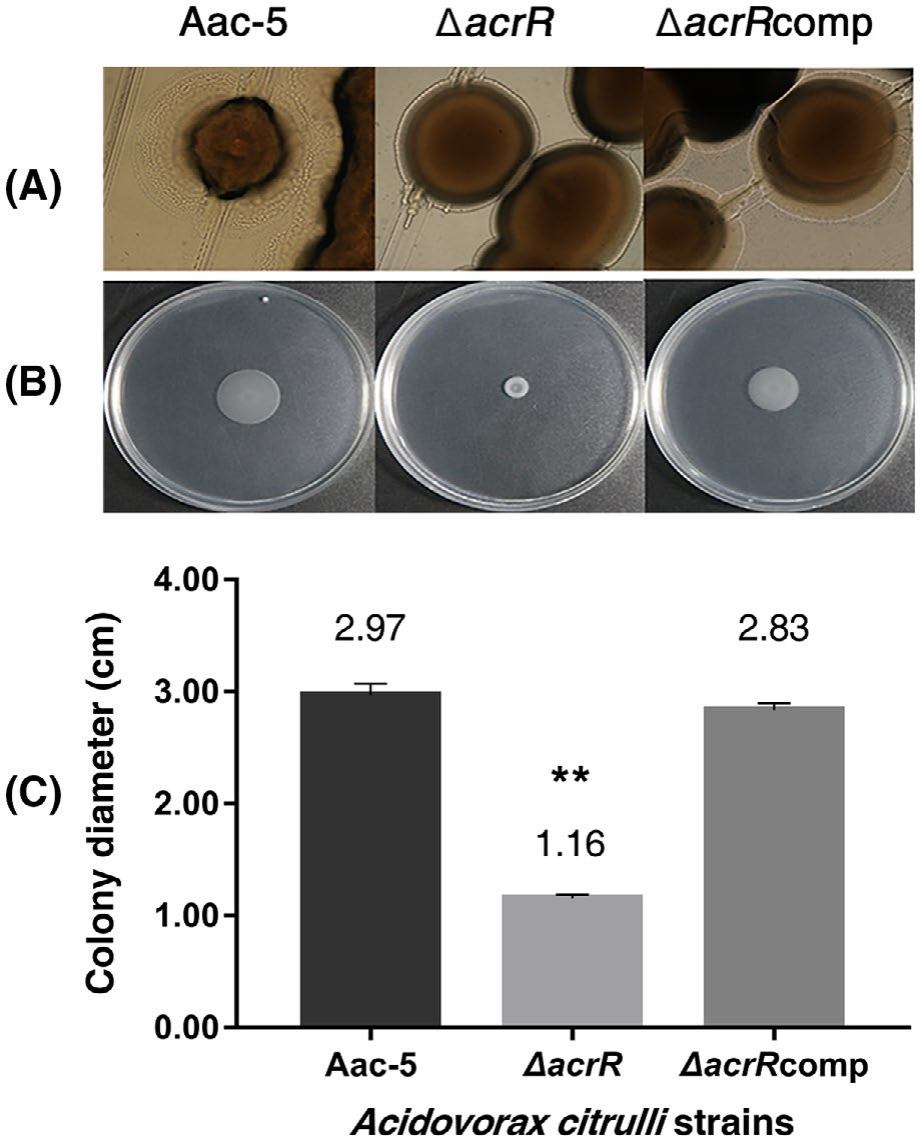
Effect of *acrR* on twitching and swimming motilities of *Acidovorax citrulli*. (a) The wild‐type strain Aac‐5 and the complementation strain ∆*acrR*comp produced typical haloes around bacterial colonies, indicating bacterial motility via twitching. The mutant strain ∆*acrR* produced few or no haloes. Thirty colonies were observed for each strain. (b) Comparison of swimming motility of *A. citrulli* strains 36 hr after inoculation of 10 µl each bacterial suspension to the centre of a soft basal medium plate at 28 °C. (c) Swimming motility measured by colony diameters of each strain on basal medium plate. The bars represent standard errors of the means from three experiments, and each experiment contained three replicates for each strain. **p < .01 by Student's *t* test

We compared the wild‐type strain Aac‐5 to its mutant strain ∆*acrR* and the complementation strain ∆*acrR*comp for the formation of corrugated trajectories or halos around their colonies as each bacterium migrated via twitching motility. Strain Aac‐5 produced typical corrugated haloes, while smooth haloes were produced by the ∆*acrR* strain and the ∆*acrR*comp strain (Figure 5a). These results show that the *A. citrulli* strain lost the twitching ability when its *acrR* gene was mutated.

Our assay for swimming motility revealed that the mutant strain ∆*acrR* did not spread 36 hr after inoculation of 10 µl of the bacterial suspension into the centre of a soft agar plate (0.3% agar), whereas the wild‐type strain Aac‐5 and the complementation strain ∆*acrR*comp spread to approximately one quarter of the plate (Figure 5b). When the bacterial suspension was placed on the basal medium plate, the average diameter of the drop was 1.01 cm. After 36 hr incubation at 28 °C, the average diameter of the bacterial lawn was 2.97 cm for the wild‐type strain Aac‐5, significantly larger than 1.16 cm for the mutant strain ∆*acrR*, but was similar to 2.83 cm for the complementation strain ∆*acrR*comp (*p* < .01) (Figure 5c). The fact that the size of the bacterial lawn for the mutant strain ∆*acrR* remained similar 36 hr after incubation indicated the loss of swimming motility.

**Figure 6:**
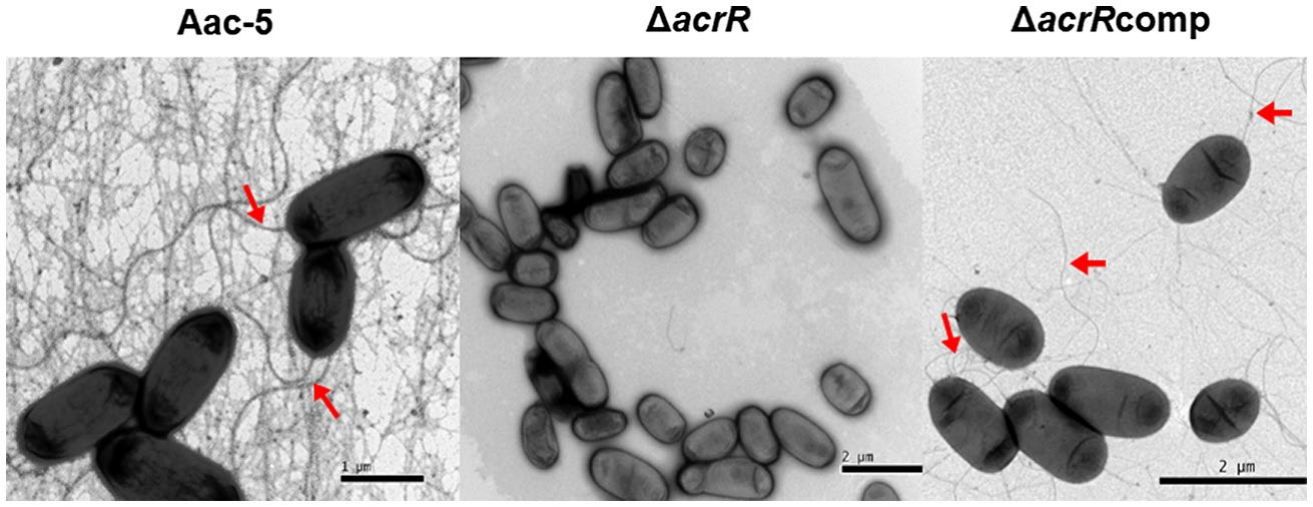
Effect of *acrR* on formation of polar flagellum of *Acidovorax citrulli*. The formation of polar flagellum observed under transmission electron microscopy. Clear filament polar flagella (indicated by arrows) were observed in the wild‐type strain Aac‐5 and complementation strain ∆*acrR*comp, but not in the mutant strain ∆*acrR*

To determine what might cause the loss of swimming motility, we compared the flagellar formation of *A. citrulli* strains. Under transmission electron microscopy, polar flagella were observed in the wild‐type and complemented strains but not in the mutant strain (Figure 6).

### 2.5 RNA‐Seq revealed that the *acrR* gene plays a role in the flagellar formation of *A. citrulli*

**Figure 7:**
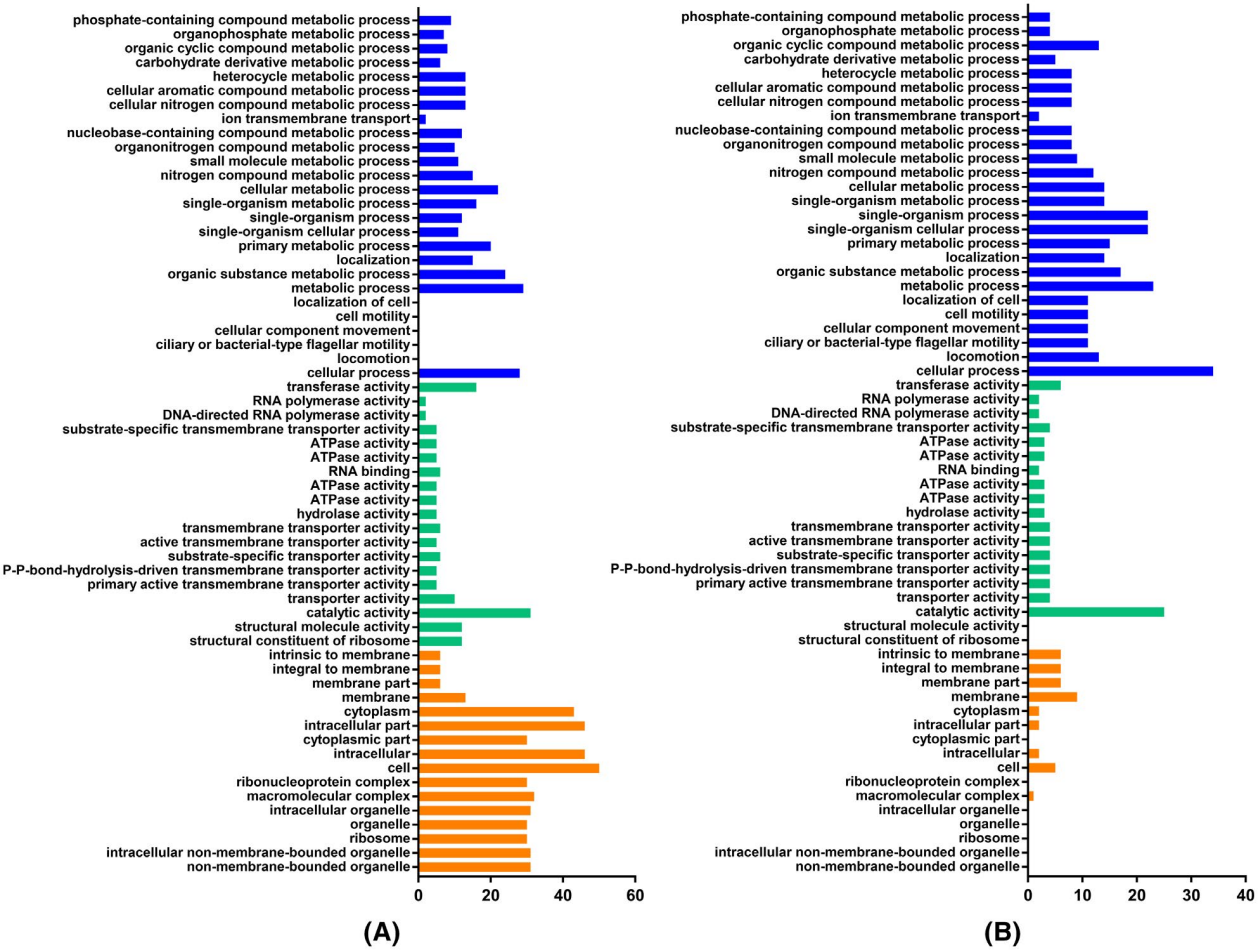
Gene ontology of highly (a) and lowly (b) differentially expressed genes between *acrR* mutant and wild‐type strains of *Acidovorax citrulli*. The *y* axis represents terms of gene ontology, which are divided into three categories: biological process indicated in blue, molecular function in green, and cellular component in orange. The *x* axis represents the number of genes

Because AcrR is identical to an annotated LuxR‐type regulator in *A. avenae* subsp. *avenae*, we explored the possible regulatory role played by AcrR by comparing the *acrR* mutant strain ∆*acrR* of *A. citrulli* with its wild‐type strain Aac‐5 through transcriptome and gene ontology analyses. A total of 394 genes were differentially expressed in the ∆*acrR* mutant compared to its wild‐type strain, including 219 highly and 175 lowly expressed genes (Table S1). The RNA‐Seq results were validated in quantitative reverse transcription PCR (RT‐qPCR) experiments with a set of 10 selected genes (Figure S1). Gene ontology analysis divided the differentially expressed genes (DEGs) into biological process, cellular component, and molecular function (Figure 7). Under the category of biological process, the DEGs related to processes of bacterial flagella and cell motility (subcategories cell motility, cellular component movement, ciliary or bacterial‐type flagellar motility and locomotion) were significantly lowly expressed, whereas the DEGs involved in cellular metabolic process were significantly highly expressed, indicating the regulatory role that *acrR* plays in the motility and metabolism of the *A. citrulli* strain Aac‐5 (Figure 7). Under the categories of cellular component and molecular function, the numbers of highly expressed genes were more than that of lowly expressed genes, especially under the category of cellular component, which includes highly expressed genes hitting 13 out of 16 subcategories (Figure 7). Taken together, the results of the gene ontology analysis were consistent with the abolished motility and increased in vitro growth ability of *acrR* mutant of *A. citrulli* Aac‐5.

**Figure 8:**
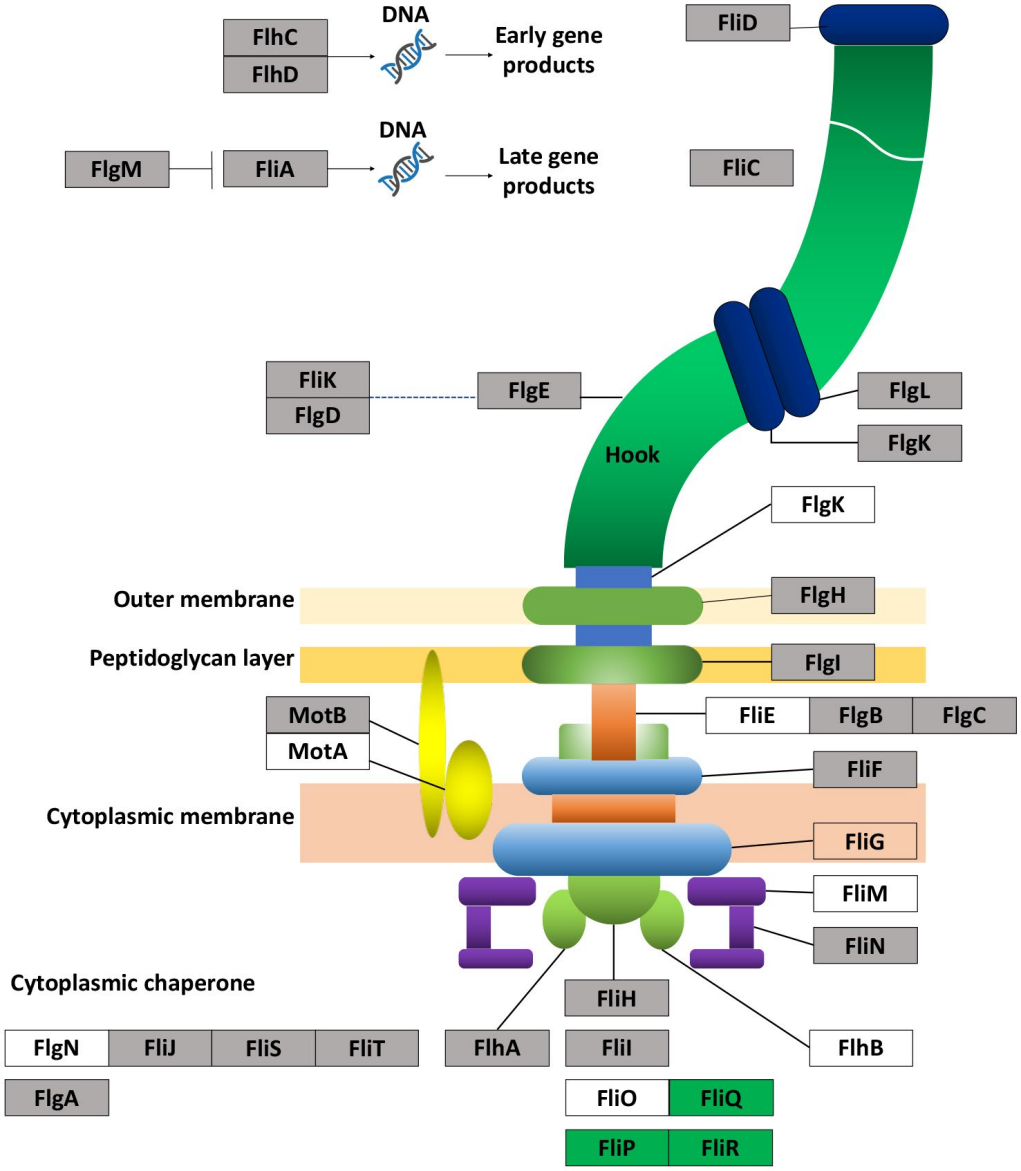
Schematic diagram of the differentially expressed genes involved in flagellar assembly. Layers of inner and outer membrane, peptidoglycan layer, C‐, MS‐, P‐, and L‐rings, motor proteins (MotAB), rotor (FlgBCF), hollow rod, hook, and distal part of flagellum are shown. Components of flagellum transport apparatus, ATPase complex, chaperones of flagellar transport, and their substrates are presented schematically**.** Highly expressed genes are shaded grey and lowly expressed genes are highlighted in green

The RNA‐Seq data also showed that deletion of the *acrR* gene affected many genes involved in flagellar biosynthesis. Kyoto Encyclopedia of Genes and Genomes (KEGG) pathway analysis revealed that 33 DEGs were involved in flagellar biosynthesis, including 3 highly and 30 lowly expressed genes (Table 3). The lowly expressed genes encode critical components for flagellar assembly, including flagellin, flagella biosynthesis proteins, flagella L‐g and P‐ring proteins located in outer membrane and peptidoglycan layer, flagella cap, hook and basal‐body rod related proteins, as well as transcriptional regulators FlhDC (Figure 8). The RNA‐Seq results were consistent with the loss of flagella formation in the mutant strain when *acrR* was deleted (Figure 6). In addition, 46 DEGs were found involved in ribosomal proteins, all of which were significantly highly expressed (Table S1).

**Table 3:** Differentially expressed genes involved in flagellar biosynthesis in *Acidovorax citrulli* Δ*acrR* strain compared to its wild‐type strain Aac‐5

Gene ID	Gene product description	logFC	*p* value
*Flagellar assembly*			
Aave_2005	Transcriptional activator FlhC	−2.77	6.64E−03
Aave_2006	Flagellar transcriptional regulator FlhD	−2.83	5.87E−03
Aave_2496	Chemotaxis protein CheW	−1.92	2.84E−03
Aave_4383	Flagellar biosynthetic protein FliR	4.68	4.69E−24
Aave_4384	Flagellar biosynthesis protein FliQ	1.97	1.12E−04
Aave_4385	Flagellar biosynthetic protein FliP	1.48	4.31E−03
Aave_4387	Flagellar motor switch protein FliN	−1.11	3.95E−02
Aave_4390	Flagellar hook‐length control protein FliK	−1.91	1.18E−04
Aave_4391	Flagellar hook‐length control protein FliJ	−2.63	2.14E−03
Aave_4392	Flagellar protein export ATPase FliI	−1.68	1.98E−03
Aave_4393	Flagellar assembly protein FliH	−1.91	2.83E−04
Aave_4395	Flagellar M‐ring protein FliF	−2.04	3.52E−05
Aave_4397	Flagellar protein FliT	−2.89	1.40E−09
Aave_4398	Flagellar export chaperone FliS	−3.13	1.88E−05
Aave_4399	Flagellar cap protein FliD	−1.34	6.45E−03
Aave_4400	Flagellin	−4.35	9.36E−22
Aave_4401	Flagellin	−2.28	4.33E−07
Aave_4413	Flagellar biosynthesis protein FlhA	−3.97	1.34E−16
Aave_4414	Flagellar biosynthesis protein FlhF	−3.29	3.55E−12
Aave_4416	RNA polymerase sigma factor FliA	−1.76	9.25E−06
Aave_4418	Flagellar biosynthesis anti‐sigma factor FlgM	−2.81	3.19E−07
Aave_4419	Flagella basal body P‐ring formation protein FlgA	−2.2	1.22E−04
Aave_4420	Flagellar basal‐body rod protein FlgB	−3.74	2.00E−05
Aave_4421	Flagellar basal body rod protein FlgC	−3.51	4.18E−06
Aave_4422	Flagellar basal body rod modification protein FlgD	−3.94	9.00E−14
Aave_4423	Flagellar hook protein FlgE	−4.35	6.65E−20
Aave_4424	Flagellar basal body rod protein FlgF	−4.39	1.60E−14
Aave_4426	Flagellar L‐ring protein FlgH	−3.55	3.19E−11
Aave_4428	Flagellar P‐ring protein FlgI	−3.7	5.46E−13
Aave_4429	Flagellar rod assembly protein/muramidase FlgJ	−3.32	1.23E−10
Aave_4430	Flagellar hook‐associated protein FlgK	−2.92	2.69E−09
Aave_4431	Flagellar hook‐associated protein 3 FlgL	−2.86	8.98E−09
Aave_4592	Flagellar motor protein MotB	−1.95	1.07E−04

Note

Gene ID refers to the locus_tag of a differentially expressed gene in *A. citrulli* Δ*acrR* strain compared to strain Aac‐5, identified by hits in a BLASTn search against the strain AAC00‐1 genome (GenBank accession number CP000512.1). FC, fold‐change

## 3 DISCUSSION

In this study, we identified a LuxR‐type transcriptional regulator AcrR in *A. citrulli*. We demonstrated that deletion of the *acrR* gene resulted in reduced virulence on watermelon seedlings, lost twitching and swimming motilities, and failure to form flagella, but increased biofilm formation and growth ability of the mutant strain compared to its wild‐type strain of *A. citrulli*. Additionally, comparative RNA profiling analysis revealed that 394 genes were differentially expressed, including 33 involved in flagellar assembly, suggesting the regulatory role AcrR plays, especially in flagella‐related functions of *A. citrulli*.

LuxR‐type regulators are known to play a role not only in the QS system but also in other biological functions such as flagellar synthesis. For example, VisN and VisR are two regulators in *Sinorhizobium meliloti* that belong to the LuxR family and act as global regulators of chemotaxis, flagellar, and motility genes (Sourjik *et al.*, 2000). As demonstrated in our study, the AcrR of *A. citrulli* either positively or negatively regulated multiple biological functions of *A. citrulli*, because the *acrR* mutant strain lost swimming motility and failed to twitch and form flagella but increased in biofilm production and growth ability.

The bacterial flagellum is an organelle for cell propulsion (Macnab, 2004). In addition, the flagellum is involved in several functions associated with bacterial pathogenicity, including biofilm formation, protein export, and adhesion (Haiko and Westerlund‐Wikström, 2013). The flagellum also serves as a virulence factor in many bacteria, including *Salmonella typhimurium*, *Escherichia coli*, *Vibrio cholera*, and *Pseudomonas aeruginosa* (Duan *et al.*, 2013). Bahar *et al. *(2011) found that an intact flagellum was required to achieve full virulence of *A. citrulli*. They observed that the *A. citrulli* strain M6 was unable to form an intact flagellum when its *fliC* gene was mutated, and the mutant was reduced in virulence and twitching motility although not in biofilm formation (Bahar *et al.*, 2011). In our study, the deletion of the *acrR* gene abolished the ability of *A. citrulli* strain Aac‐5 to form a polar flagellum, which, in turn, may have resulted in reduced swimming motility, contributing to its reduced virulence on watermelon seedlings. Our RNA‐Seq data support the role that AcrR plays in flagellar biosynthesis because 33 genes involved in flagellar assembly were differentially expressed in the *acrR* mutant strain in comparison with the wild‐type strain (Table 3).

The lack of flagella could lead to enhanced growth ability in prokaryotes. *Pyrococcus furiosus* DSM363 exhibited an enhanced growth ability when its flagella were absent (Lewis *et al.*, 2015). The transcription regulator SwrA stimulates the transcription of the genes for σ^D^, which controls when *Bacillus subtilis* cells enter into the motile state with expression of flagellum biosynthesis genes from a state of growth as long and nonmotile chains (Kearns and Losick, 2005). For growth and maintenance on the host, bacteria may reduce or eliminate flagellar expression (Chaban *et al.*, 2015). In our study, the deletion of the *acrR* gene not only eliminated the biosynthesis of flagellum of *A. citrulli* Aac‐5 strain but also enhanced its growth ability, whereas the complementation strain showed similar growth ability to the Aac‐5 strain, indicating that AcrR may regulate the Aac‐5 switch between the growth and motility stages. The fact that 46 ribosomal genes were highly expressed in the mutant strain compared to the wild‐type one (Table S1) suggests that these genes may contribute to the enhanced growth ability of the mutant strain.

Flagellar synthesis is a strictly hierarchical process in which more than 50 genes are involved (Wang *et al.*, 2015). These genes are organized in multiple operons that can be divided into three classes based on the sequential order of the process: early (I), intermediate (II), and late (III) (Aldridge and Hughes, 2002; Osterman *et al.*, 2015). Operon *flhDC* codes for transcription factor FlhDC, a master flagellar regulator. It is considered the sole class I transcription unit and is responsible for transcriptional activation of all structural and other regulatory components of the flagellar machinery (Fitzgerald *et al.*, 2014). In *A. citrulli*, we found that among the 33 differentially expressed genes associated with flagellar assembly, *flhC* and *flhD* genes were lowly expressed with 2.77‐ and 2.83‐log fold change, respectively, suggesting that the early stage of flagellar synthesis is regulated by AcrR. The class II sigma‐factor *fliA* gene regulates the transition from early to late‐stage flagellar gene expression (Fitzgerald *et al.*, 2014; Osterman *et al.*, 2015). Along with its cognate *flgM* gene, an anti‐sigma factor, *fliA* was significantly lowly expressed in the *acrR* mutant, which in turn down‐regulated its downstream class III flagellar synthesis genes *flgKL*, *fliDST*, *motB*, and *cheW* (Table 3).

Why the three genes, *fliP*, *fliQ*, and *fliR*, which code for FliP, FliQ, and FliR, respectively, in flagellar biosynthesis were highly expressed in the *acrR* mutant in comparison to the wild‐type strain is unclear. FliP, FliQ, and FliR are components of the flagellar secretion apparatus that anchors at the cell membrane (Ward *et al.*, 2018) and belongs to the flagellar type III secretion system (fT3SS). They reside within the MS‐ring, a subdomain of the hook‐basal‐body, which is located within the cytoplasmic membrane (Figure 8) (Zhuang and Shapiro, 1995; Fan *et al.*, 1997). Assembly of the flagellum begins with the MS‐ring, comprising several transmembrane proteins, belonging to the fT3SS and inserted into the cellular membrane (Macnab, 2004). Because the *acrR* gene (nucleotides 4874913 to 4875542) is located at the 3′ flanking region on the complementary strand of the operon *fliRQP* (nucleotides 4875558 to 4877416) in the genome of *A. citrulli* strain AAC00‐1, the deletion of the *acrR* gene might not affect the promoter region of the operon *fliRQP*. The fact that all the differentially expressed flagellar assembly related genes except *fliR*, *fliQ*, and *fliP* were lowly expressed in the *acrR* mutant suggests that the *fliR*, *fliQ*, and *flip* genes might be regulated by other factors when *acrR* is absent.

Biofilm formation is crucial for the virulence of some plant pathogenic bacteria (Dow *et al.*, 2003; Fujishige *et al.*, 2006). The phytopathogenic bacterium *Xanthomonas citri* showed reduced disease symptoms when it was unable to form a biofilm (Rigano *et al.*, 2007). In contrast, our study revealed that biofilm formation of the *acrR* mutant of *A. citrulli* was not decreased but increased significantly compared to the wild‐type strain Aac‐5, while the virulence of the mutant was reduced. This result is in agreement with previous findings that some of the group II strains of *A. citrulli* are not able to form a biofilm, while the group I strain M6 is able to form a biofilm (Bahar *et al.*, 2009; Chen *et al.*, 2009), suggesting potential differences in the trajectory of biofilm formation processes between some of the strains that belong to the two groups of *A. citrulli* strains.

Twitching is a process that contributes to the adherence of bacterial cells to surfaces and colonization on bacterial hosts (Mattick, 2002; Craig *et al.*, 2004). It is also required for the virulence and biofilm formation of *A. citrulli* (Bahar *et al.*, 2009). When the *acrR* gene was mutated in Aac‐5 of *A. citrulli*, no twitching motility was observed and the virulence was reduced, suggesting that *acrR* is important for twitching motility in *A. citrulli*, which, in turn, contributes to the virulence of the bacterium. An interesting finding of this study is that the *acrR* mutant lost the ability to twitch but increased its ability to form a biofilm compared to the wild‐type strain Aac‐5. This is different from previous findings that *A. citrulli* group I strain M6 and its mutants lacked the ability to twitch and were also decreased in biofilm formation (Bahar *et al.*, 2009; Rosenberg *et al.*, 2018). It is possible that the twitching motility and biofilm formation are regulated differently in group I and II strains, and the global transcriptional regulator AcrR may regulate many genes, including the ones involved in biofilm formation.

In summary, the *acrR* gene contributes to the virulence of *A. citrulli* strain Aac‐5, either directly and/or indirectly though positive regulation of the twitching and swimming motilities and flagellar formation and negative regulation of the biofilm formation. Additionally, our transcriptomic analysis revealed that the *acrR* gene also positively regulates flagellar assembly in *A. citrulli,* supporting the role that AcrR plays in flagellar biosynthesis*.* Because AcrR contains a receptor domain at the N‐terminus, possibly the AcrR senses or interacts with certain signals that affect multiple biological functions, including virulence and flagellar formation of *A. citrulli*. Future research is needed to identify such signals and elucidate the molecular mechanisms behind the regulation of *A. citrulli* by AcrR for the development of effective control strategies to combat this important bacterial pathogen.

## 4 EXPERIMENTAL PROCEDURES

### 4.1 Bacterial strains, plasmids, growth conditions, and primer design

The bacterial strains and plasmids used in this study are listed in Table 1. *A. citrulli* strains were grown in King's B (KB) broth or on a KBA plate (KB containing agar at 15 g/L) with appropriate antibiotics and at 28 °C. *E. coli* strains were grown in Luria Bertani medium at 37 °C. The antibiotics used were ampicillin (Ap), kanamycin (Km), and gentamicin (Gm) at concentrations of 100 µg/ml for Ap and 50 µg/ml for the other antibiotics. The primer pair acrR‐F/R was designed based on *acrR* gene in AAC00‐1 genome (GenBank accession number CP000512.1), while primer pairs LacR‐F/R and RacrR‐F/R were designed based on upstream and downstream sequences of the *acrR* gene (Table 1). Primers GmF and GmR were designed based on gentamicin cassette (Table 1). All primers used in this study were designed using the free online program Primer 3.0 (http://www.simgene.com/Primer3).

### 4.2 Construction of the *acrR* mutant and its complemented strain

The *acrR* gene was deleted by homologous double recombination as described previously (Wang *et al.*, 2016). Briefly, the 472‐bp upstream and 541‐bp downstream sequences of the *acrR* gene were amplified from the wild‐type strain Aac‐5 using the LacrR‐F/LacrR‐R and RacrR‐F/RacrR‐R primers (Table 3). After confirmation by sequencing, the PCR fragments were digested by appropriate restriction enzymes and ligated into pK18 mobsacB to create the plasmid pK18‐*acrR*‐Up&Down (Table 2). The plasmid was digested by *Bam*HI and *Sal*I, and a Gm gene cassette (855 bp) was inserted between the *Bam*HI and *Sal*I sites to create plasmid pK18‐*acrR*Gm (Table 3). The plasmid was then introduced from *E. coli* DH5α into the *A. citrulli* strain Aac‐5 by triparental conjugation using pRK600 as a helper plasmid to create the *acrR* mutant strain Δ*acrR* (Table 2). Transconjugants were screened on KBA supplemented with 10% sucrose and antibiotics (Ap and Gm). The presence of the Gm cassette in the transconjugants was confirmed by PCR and sequencing of the amplified PCR product using the primer pair GmF/GmR (Table 1). The absence of the *acrR* gene in the transconjugants was confirmed by the lack of PCR product using primer pair acrR‐F/acrR‐R.

To generate a complementation strain of Δ*acrR*, the *acrR* gene in Aac‐5 was amplified using primers acrR‐F and acrR‐R (Table 1). The PCR product was digested with *Hin*dIII and *Bam*HI and cloned into pBBR1MCS‐2 to generate pBBR‐acrR, which was transferred into the mutant strain ∆*acrR* by triparental conjugation (Table 2). The successful transconjugant, named ∆*acrR*comp, was identified through screening on KBA (amended with Ap, Km, and Gm; Table 2). All obtained plasmids and *A. citrulli* strains were confirmed by PCR and DNA sequencing.

### 4.3 Virulence assays

The virulence of the *A. citrulli* strains was tested on 3‐week‐old watermelon seedlings (*Citrullus lanatus* 'Jingxin#6', provided by the Beijing Academy of Agriculture and Forestry Sciences, Beijing, China). The virulence assay was performed, and the DI was calculated as previously described (Wang *et al.*, 2016). Briefly, *A. citrulli* strains were grown in KB broth and their OD_600_ was adjusted to 0.6 (approximately 10^8^ cfu/ml). Two hundred millilitres of each bacterial suspension was sprayed onto watermelon seedlings grown at 28 °C in a growth chamber with 90% relative humidity. Disease symptoms were evaluated at the eighth day after inoculation using a disease severity scale: 0 for no symptoms; 1, 3, 5, and 7 for necrotic lesions on approximately 25%, 50%, 75%, and 100% of the leaves, respectively; and 9 for complete death of the seedling. The disease index was calculated based on the formula $DI=\sum \left(A\times B\right)\times 100/\sum C\times 9$, where *A* is the disease scale, *B* is the number of seedlings in each disease scale, and *C* is the total number of seedlings in each treatment. For each *A. citrulli* strain, six watermelon seedlings in three pots were inoculated in each experiment, and the experiment was repeated three times.

### 4.4 In planta growth assays

The growth ability of *A. citrulli* strains in 2‐week‐old watermelon cotyledons (the same cultivar as the one used in virulence assay) was determined using the method of Johnson *et al. *(2011), with modifications. Briefly, *A. citrulli* was grown overnight in KB broth at 28 °C to an OD_600_ of 0.8. The bacterial cells were then washed three times with sterile water and adjusted to 10^4^ cfu/ml with sterile water to make the bacterial inoculum. One millilitre of the inoculum was injected into 30 watermelon cotyledons using sterile syringes. Six injected cotyledons, each inoculated with the *A. citrulli* strain or water, were collected at 1, 24, 48, 72, and 96 hai, respectively. One 3‐mm disc from each cotyledon and six discs in total were collected and homogenized in Lysing Matrix A tubes (MP Biomedicals Co., Ltd) containing 1 ml of sterile water using MP FastPrep‐24^T^ 5^G^ (MP Biomedicals Co., Ltd). The lysates were serially diluted with sterile water and plated on KBA plates. The plates were incubated at 28 °C for 48 hr. The growth of colonies on the KBA plates was counted as a measurement of the population of *A. citrulli* strains growing in watermelon cotyledons. The experiment was repeated three times.

### 4.5 Assay for swimming and twitching motilities and observation of flagella by transmission electron microscopy

Swimming and twitching motilities of the *A. citrulli* strains were determined using the methods of Wang *et al. *(2016). For swimming motility, *A. citrulli* strains were incubated in KB broth and their OD_600_ was adjusted to 0.6. Five microlitres of each cell suspension was placed into the centre of a basal medium plate containing 0.3% agar and incubated for 36 hr at 28 °C. The diameter of the colony on each plate was then measured. For twitching motility, 10 μl of *A. citrulli* strains were placed on the KBA plates containing ampicillin and incubated for 72 hr. The twitching motility was visualized using a BX63 microscope (Olympus). Colonies with twitching motility were characterized by the formation of corrugated trajectories or haloes around the colonies. In both swimming and twitching motility assays, at least nine plates were inoculated by each strain in each experiment, and the experiment was repeated three times.

Flagella of each *A. citrulli* strain were observed using the method described by Wang *et al. *(2016). Briefly, each *A. citrulli* strain was grown on the basal medium that was used in the swimming motility assay for 36 hr. Ten microlitres of sterile water was placed on top of each colony for 2 min, and then covered by a 200‐mesh copper grid for 1 min. The copper grids were negatively stained with 1% uranyl acetate three times, each time for 30 s, before they were dried on sterile filter paper. The presence or absence of polar flagella in each *A. citrulli* strain was examined under transmission electron microscopy.

### 4.6 Biofilm and growth ability assays

The effect of *acrR* deletion on biofilm formation was determined both qualitatively and quantitatively, and the growth rate of the *A. citrulli* strains was measured as described by Wang *et al. *(2016). Briefly, overnight cultures of *A. citrulli* strains were adjusted to an OD_600_ of 0.1. One hundred microlitres of each cell suspension was added to 10 ml KB broth in a glass flask and incubated at 28 °C for 7 days without agitation. Two millilitres of 0.1% (wt/vol) crystal violet was then added to the flask and incubated for 2 hr. After the liquid had slowly been poured off, the flasks were rinsed with distilled water and fixed by heating at 80 °C for 30 min. To quantify the biofilm formation, the stained biofilm was solubilized by 5 ml ethanol for 12 hr, and the OD_580_ of the stained suspension was measured with a spectrophotometer (Biophotometer, Eppendorf). Three biological replicates were performed for each *A. citrulli* strain in each experiment, and the experiment was repeated three times.

To measure bacterial growth in vitro, 2 μl of cell suspension for each *A. citrulli* strain at OD_600_ of 0.3 was diluted 100‐fold with 198 μl KB broth in a well of a 96‐well plate. The plate was incubated at 28 °C with shaking at 220 rpm. The OD_600_ of the suspension was measured every 2 hr for 72 hr by Bioscreen C (FP‐1100‐C, Oy Growth Curves Ab Ltd).

### 4.7 RNA isolation and quantitative reverse transcription PCR analysis of gene expression

RNA was isolated from the *A. citrulli* strains using the method of Wang *et al. *(2016). Briefly, *A. citrulli* was grown in KB broth at 28 °C overnight to an OD_600_ of 0.8. The bacterial cells were then washed twice with sterilized water and their RNA was extracted using a bacterial RNAout kit (Tiandz) based on the manufacturer's instructions. cDNA was synthesized based on the method of Wang *et al. *(2016) using a FastQuant RT kit (TianGen). RT‐qPCR analysis was carried out using primers designed for 10 DEGs (Table S2). cDNA was used as a template with SYBR Green added in the PCR, and relative levels of gene expression were determined as previously described (Wang *et al.*, 2016). Three biological replicates were established in each experiment, and the experiment was repeated three times.

### 4.8 RNA‐Seq library construction and sequencing

A total of 3 μg of RNA per *A. citrulli* strain was used as input material for subsequent RNA sample preparations. The RNA sequencing libraries were constructed and sequenced commercially by Novogene Co., Ltd using NEBNext Ultra RNA Library Prep Kit for Illumina (NEB), and index codes were added to attribute sequences to each sample. The clustering of the index‐coded samples was performed on a cBot Cluster Generation System using a TruSeq PE Cluster Kit v. 3‐cBot‐HS (Illumina). After cluster generation, the library preparations were sequenced on an Illumina HiSeq 2500 platform, and 100‐bp paired‐end reads were generated.

### 4.9 RNA‐Seq data analysis

The sequencing data were analysed commercially by Novogene Co., Ltd. Briefly, analysis for differential gene expression between the wild‐type strain Aac‐5 and the *acrR* mutant strain Δ*acrR* (three biological replicates per strain) was performed using the DESeq R package (v. 1.10.1). DESeq provides statistical routines for determining differential expression in digital gene expression data using a model based on the negative binomial distribution. The resulting *p* values were adjusted using the Benjamini and Hochberg's approach for controlling the false discovery rate. Genes with an adjusted *p* value <.05 found by DESeq were assigned as differentially expressed (Pan *et al.*, 2018).

Gene ontology enrichment analysis of the DEGs was implemented by the goseq R package, in which the gene length bias was corrected. GO terms with a corrected *p* value less than .05 were considered significantly enriched by the DEGs (Pan *et al.*, 2018).

### 4.10 Statistical analysis

Statistical analysis was performed using the Student's *t* test in Excel 2010 software (Microsoft Inc.). Differences were considered statistically significant if *p* < .01.

## CONFLICT OF INTEREST

The authors declare that the research was conducted in the absence of any commercial or financial relationships that could be construed as a potential conflict of interest.

## AUTHOR CONTRIBUTIONS

W.G., T.W., and T.Z. designed the research. W.G. wrote the paper. T.W., W.G., E.T., and B.L. executed the experiments. W.G., T.W., and Y.Y. performed the data analyses. Q.H. revised the manuscript. All authors read and approved the final manuscript.

## Supporting information


**FIGURE S1** Relative expression of selected genes by RT‐qPCR. The bacterial strains were incubated under the same condition as those for the RNA‐Seq experiment (three biological replicates per strain). The *x* axis represents log_2 _(fold‐change) of each gene in *acrR* mutant compared to its wild‐type strain Aac‐5

**TABLE S1** Differentially expressed genes between the *Acidovorax citrulli acrR* mutant strain and its wild‐type strain Aac‐5

**TABLE S2** Primers used for RT‐qPCR

## Data Availability

The data that support the findings of this study are available from the corresponding author upon reasonable request.
