# Supplementary figures and images for: A LuxR‐type regulator, AcrR, regulates flagellar assembly and contributes to virulence, motility, biofilm formation, and growth ability of Acidovorax citrulli
Source: Mol Plant Pathol. 2020 Jan 14;21(4):489–501. doi: 10.1111/mpp.12910 (PMC7060138; doi:10.1111/mpp.12910)

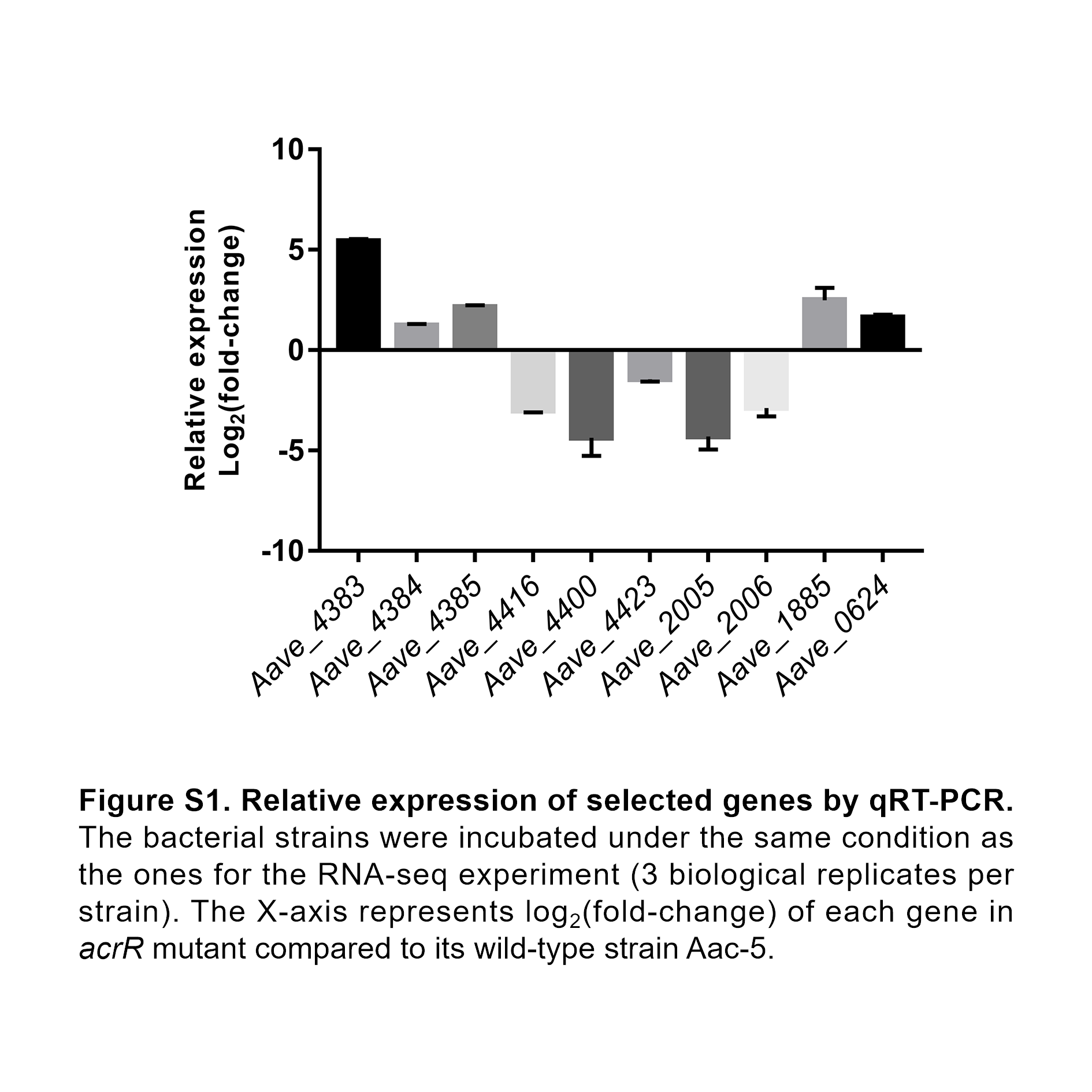

Supplement: Supplementary file 1 — FIGURE S1 Relative expression of selected genes by RT‐qPCR. The bacterial strains were incubated under the same condition as those for the RNA‐Seq experiment (three biological replicates per strain). The x axis represents log2 (fold‐change) of each gene in acrR mutant compared to its wild‐type strain Aac‐5 [file MPP-21-489-s001.tif]
