# Supplementary material for: A LuxR‐type regulator, AcrR, regulates flagellar assembly and contributes to virulence, motility, biofilm formation, and growth ability of Acidovorax citrulli
Source: Mol Plant Pathol. 2020 Jan 14;21(4):489–501. doi: 10.1111/mpp.12910 (PMC7060138; doi:10.1111/mpp.12910)
Supplement: Supplementary file 3 — TABLE S2 Primers used for RT‐qPCR [file MPP-21-489-s003.docx]

Table S2. Primers used for RT-qPCR

| Primers | Sequence (5’–3’) | |
| --- | --- | --- |
| 4383F | | GTGCTGTGGCCGTTCCTG |
| 4383R | | GCTGACGACGGGCTGGT |
| 4384F | | ACCTCCCAAATGGTCCTGACG |
| 4384R | | GAGCTTGGGCACGAAGGC |
| 4385F | | CATGGCTGTGGCGGTCG |
| 4385R | | CAGCGCCGTGAAGAACAGC |
| 4416F | | GCATTGATCCGCCAGCACG |
| 4416R | | CCTGCGTCACCTCGTAGCG |
| 4400F | | ACGGCTCCTTCGGCTCG |
| 4400R | | GGAGGCCGAAGCCGTCA |
| 4423F | | CTCAACGCGGCCAGCAAG |
| 4423R | | CATTGGTTCCGCCCGCG |
| 2005F | | GCGTCCTGAACGAATCCAAGC |
| 2005R | | GCTGGCCCTTGGAAGGC |
| 2006F | | AGGCCGAAGCCGTGTTCC |
| 2006R | | GCAGGCTCCAGACGAGGTT |
| 1885F | | TGCCCGCCCTTCCCATC |
| 1885R | | CCTTCTCGCTGGGCTGCA |
| 0624F | | GCAAAGTCTCCCGCCAATAACG |
| 0624R | | GGAAGAGGCCCAGGACAGG |
